# Comprehensive transcriptomics and metabolomics analyses reveal that hyperhomocysteinemia is a high risk factor for coronary artery disease in a chinese obese population aged 40–65: a prospective cross-sectional study

**DOI:** 10.1186/s12933-023-01942-0

**Published:** 2023-08-24

**Authors:** Chong-Yu Zhang, Ru-Qin Xu, Xiao-Qiao Wang, Lin-Feng Sun, Pei Mo, Ren-Jie Cai, Xiao-Zhen Lin, Cheng-Feng Luo, Wen-Chao Ou, Lie-Jing Lu, Yun Zhong, Jia-Yuan Chen

**Affiliations:** 1 Department of Cardiology, Guangzhou Institute of Cardiovascular Disease, Guangdong Key Laboratory of Vascular Diseases, The Second Affiliated Hospital, Guangzhou Medical University, Guang Zhou, China; 2 Department of Anesthesiology, the Second Affiliated Hospital of Guangzhou Medical University, Guang Zhou, China; 3 Department of Anesthesiology, Sun Yat-Sen Memorial Hospital, Sun Yat-Sen University, Guang Zhou, China; 4 No.250 Changgang Road, Guangzhou, Haizhu district China

**Keywords:** Metabolomics, Obese, Coronary artery disease, Hyperhomocysteinemia, S-adenosylhomocysteine, Cohort, ROC analysis

## Abstract

**Background:**

Clinical observations suggest a complex relationship between obesity and coronary artery disease (CAD). This study aimed to characterize the intermediate metabolism phenotypes among obese patients with CAD and without CAD.

**Methods:**

Sixty-two participants who consecutively underwent coronary angiography were enrolled in the discovery cohort. Transcriptional and untargeted metabolomics analyses were carried out to screen for key molecular changes between obese patients with CAD (CAD obese), without CAD (Non-CAD obese), and Non-CAD leans. A targeted GC-MS metabolomics approach was used to further identify differentially expressed metabolites in the validation cohorts. Regression and receiver operator curve analysis were performed to validate the risk model.

**Results:**

We found common aberrantly expressed pathways both at the transcriptional and metabolomics levels. These pathways included cysteine and methionine metabolism and arginine and proline metabolism. Untargeted metabolomics revealed that S-adenosylhomocysteine (SAH), 3-hydroxybenzoic acid, 2-hydroxyhippuric acid, nicotinuric acid, and 2-arachidonoyl glycerol were significantly elevated in the CAD obese group compared to the other two groups. In the validation study, targeted cysteine and methionine metabolomics analyses showed that homocysteine (Hcy), SAH, and choline were significantly increased in the CAD obese group compared with the Non-CAD obese group, while betaine, 5-methylpropanedioic acid, S-adenosylmethionine, 4-PA, and vitamin B2 (VB2) showed no significant differences. Multivariate analyses showed that Hcy was an independent predictor of obesity with CAD (hazard ratio 1.7; 95%*CI* 1.2–2.6). The area under the curve based on the Hcy metabolomic (HCY-Mtb) index was 0.819, and up to 0.877 for the HCY-Mtb.index plus clinical variables.

**Conclusion:**

This is the first study to propose that obesity with hyperhomocysteinemia is a useful intermediate metabolism phenotype that could be used to identify obese patients at high risk for developing CAD.

**Supplementary Information:**

The online version contains supplementary material available at 10.1186/s12933-023-01942-0.

## Background

Coronary artery disease (CAD) is a main cause of heart disease-related death, and the prevalence of CAD is increasing because of increases in obesity and diabetes [1]. Clinical evidence has revealed that obesity is an independent risk factor for cardiovascular diseases [2]. Obesity is defined as excessive fat accumulation or abnormal distribution, and it is essentially a chronic endocrine metabolic disease. Recent studies demonstrated that there is significant heterogeneity and various metabolic phenotypes among obese individuals, such as metabolically abnormal obese(thought as healthy obese) and metabolically healthy obese, and the potential risk for CAD differs between these obesity subphenotypes [3, 4]. However, it remains controversy about the metabolic and cardiovascular events risk of healthy obese individuals over time [5]. Hence, fast growing studies focus on characterizing the subset of obese individual with or without CAD, and the differences in metabolic profiles in Non-CAD obese compared to Non-CAD lean control.

Recent studies have demonstrated that metabolically abnormal obesity is closely associated with poor cardiovascular outcomes. Patients with obesity with two or more cardiovascular risk factors, including increased waist circumference (WC), high C-reactive protein, and low high-density lipoprotein cholesterol (HDL-C), had a higher risk of cardiovascular mortality [6]. Hwang et al. showed that excess visceral fat deposition is associated with increased metabolic abnormality and risk of mortality [7]. However, measuring entire visceral fat using computed tomography may not be clinically practical. Isabelle et al. reported that the hypertriglyceridemic-waist phenotype, which is classified by increased fasting triglyceride (TG) levels and WC, presents an increased risk for CAD compared with low TG levels and WC (odds ratio of 3.6) [8]. Therefore, early identification of patients with obesity who are at high risk for CAD may improve patient survival, guide tailored therapy, and provide a better understanding of the risk stratification and consequences of obesity.

RNA sequencing (RNA-seq) is a transcriptome sequencing technology that identifies all RNA molecules with coding ability including mRNA, small RNA and noncoding RNA from the transcriptome through high-throughput next-generation DNA sequencing (NGS) technologies which allow RNA analysis through cDNA sequencing at massive scale [9]. Metabolomics, an emerging analytical technique used in the systemic determination of metabolite profiles in biofluids, cells and tissues, is routinely applied as a tool for investigating biomarker for a specific disease [10]. Marked metabolic disturbances are typically present in obese individuals. Metabolomics is useful for understanding metabolic changes in obesity-related cardiovascular diseases at the individual level [11]. Nevertheless, few studies have assessed the differences in transcriptomics and metabolomics profiling between CAD obese and Non-CAD obese individuals, and also few studies revealed metabolic differences between Non-CAD obese and Non-CAD lean individuals. The present study used exosomes RNA sequencing (RNA-seq) and GC-MS-based untarget metabolomic to initially determine the differences of transcriptomics and metabolomic profile in the CAD obese, Non-CAD obese and Non-CAD leans. We then applied target metabolomic approaches aimed at further characterizing the intermediate metabolism phenotypes associated with CAD obese and Non-CAD obese individuals and improving the ability to identify obese individuals at high risk for CAD beyond the traditional risk factors,

## Methods

### Study design and study population

Human clinical samples and data were collected from the Department of Cardiovascular Intern Medicine, the Second Affiliated Hospital of Guangzhou Medical University (GMU). All experiments were conducted in accordance with the study protocol approved by the Institutional Ethics Committee of the Second Affiliated Hospital of GMU (2016-ks-02; 2023-BKS-ks-01), and informed consent was obtained from all participants.

The inclusion criteria were as follows: subjects aged 40–65 years old with chest symptoms admitted to the hospital and who received coronary angiography; no CAD history; and a stable body weight for six months prior to the study. For Chinese population, the cutoffs of body mass index (BMI) for obesity, overweight and normal weight were ≥ 28 kg/m2, 24-27.9 kg/m2, and 18.5–23.9 kg/m2 respectively according to the China Obesity Working Group classification [12]. Participants were classified into the following three subgroups: CAD obese, Non-CAD obese, and Non-CAD lean.

#### CAD obese group: Body mass index (BMI) ≥28 kg/m^2^

The presence of CAD was confirmed using coronary angiography (CAG), and CAD was defined as one or more lesions leading to a more than 50% lumen stenosis of any of four vessels (right coronary, left main coronary, left circumflex and left anterior descending) [13].

#### Non-CAD obese group: BMI ≥28 kg/m^2^

The absence of CAD was confirmed using CAG.

#### Non-CAD lean group: BMI 18.5–24.9 kg/m^2^

The absence of CAD was confirmed using CAG.

The exclusion criteria were as follows: patients with signs, symptoms, or clinical history of heart failure, pericardial or valve disorders, cerebrovascular or pulmonary diseases, serious renal or liver disorders, malignant tumors, and drug abuse.

For the prospective validation study, another 59 consecutive patients were prospectively enrolled independently and divided into the Non-CAD obese and CAD obese groups. The inclusion and exclusion criteria were the same as above.

### Sample collection

Blood was collected in the morning prior to breakfast. The blood samples were placed into EDTA tubes and centrifuged at 4,000 rpm at 4℃ for 10 min to separate the plasma. The separated samples were stored at -80℃ until later use.

### Exosome purification

Three samples were randomly selected from each group for transcript analysis. The process of exosome purification was described previously, with minor modifications. Plasma samples (10 mL) were added into conical tubes and centrifuged at 1000 g at 4℃ for 15 min to remove cell fragments and lipids. The serum was further centrifuged at 1500 rpm at 4℃ using the exosomes isolation kit ExoQuick (System Bioscience), and the residual supernatant was carefully aspirated. The precipitate containing the exosomes was resuspended in 200 uL PBS and stored at -80℃. Total RNA isolation was performed using the QIAzol Lysis Reagent (Qiagen, Germany).

### Small RNA isolation

Adding 700uL of QIAzol Lysis Reagent to the exosome’s suspension before then with 140 µL of chloroform/isoamyl alcohol (24:1), the mixture was then incubated at room temperature for 2 ~ 3 min. The sample was then centrifuged at 12,000×g for 8 min at 4 ℃. Next, the entire sample was pipetted into a spin column placed in a 2 mL collection tube. The RNeasy MinElute spin column was transferred into a new 2 mL collection tube. The RNeasy MinElute spin column was loaded with the sample, and RNase-free water was added directly to the center of the spin column membrane. After a 1-minute incubation at room temperature, the column was centrifuged at full speed to elute the RNA.

### Library construction and sequencing for mRNA and lncRNA

DNase was used to remove DNA contamination from the RNA samples. Subsequently, cDNA libraries were synthesized using the TruSeq RNA Sample Prep Kit v2 (Illumina, USA) following standard protocols, starting from 300 ng of total RNA. The pre-prepared first-strand and second-strand synthesis reaction mixture were added to the fragmented RNAs to synthesize cDNAs. Next, a Polymerase Chain Reaction (PCR) reaction system was set up to amplify the cDNAs. Following PCR amplification, single-stranded PCR products were generated via denaturation. Single-stranded circle DNA molecules were then replicated via rolling cycle amplification, resulting in the creation of DNA nanoballs (DNBs) which contains multiple copies of the target DNA. Sufficient quality DNBs were then loaded into patterned nanoarrays using high-intensity DNA nanochip technique and sequenced through combinatorial Probe-Anchor Synthesis (cPAS).

### RNA-seq transcript analysis

#### Data filtering

The sequencing data was filtered with SOAPnuke by removing adapters and low-quality reads. Afterwards, clean reads were obtained and stored in FASTQ format. The subsequent analysis and data mining were performed using Dr. Tom multi-omics data mining system (https://biosys.bgi.com). Clean reads were mapped to the reference genome using HISAT2 (Version 2.1.0). Ericscript (v0.5.5) and rMATS (V3.2.5) were used to detect fusion genes and differential splicing genes (DSGs), respectively.

### RNA identification and differential expression analysis

Bowtie2 was applied to align the clean reads to the gene set. The expression level of the gene was calculated by RSEM (v1.3.1). The heatmap was drawn by pheatmap (v1.0.8) according to the gene expression difference in different samples. The mRNA and lncRNA data were aligned in a NONCODE database [14]. We used transcripts per million (TPM) for within-sample normalization for the RNA-seq data. Differential expression analysis was performed using DESeq2 (v1.4.5) with Q value ≤ 0.05 (or FDR ≤ 0.001).

### Gene annotation

To gain insights into the changes of phenotype, Gene Ontology (GO) and Kyoto Encyclopedia of Genes and Genomes (KEGG) enrichment analysis on annotated differentially expressed genes were performed using Phyper-based Hypergeometric test. The significant levels of terms and pathways were corrected using a Q value with a rigorous threshold (Q value ≤ 0.05). This correction accounts for multiple testing and helps identify only the most relevant and significant terms and pathways associated with the observed changes in phenotype.

### Metabolite extraction

To extract 100 µL metabolite samples, 300 µL of precooled methanol and acetonitrile (2:1, v/v) was directly added using an internal standard mix of L-Leucine-d3, L-Phenylalanine (13C9, 99%), L-Tryptophan-d5, and Progesterone-2,3,4-13C3 as quality control (QC). The samples were vortexed for 1 min, inclubated for 2 h at -20 °C, and centrifuged for 20 min at 4,000 rpm. The supernatant was transferred for vacuum freeze drying. To prepare the metabolites for liquid chromatography with tandem mass spectrometry (LC-MS) analysis, the sample was resuspended in 150 µL of 50% methanol and then centrifuged for 30 min at 4,000 rpm. The resulting supernatants were subsequently placed into autosampler vials. QC samples were made by combining an equivalent volume from every sample, and the reproducibility of the entire LC-MS analysis was assessed.

### Metabolite detection

A Waters 2D UPLC (Waters Corp., USA) was used to analyze the samples, followed by a heated electrospray ionization (HESI) source for coupling them to a Q Exactive mass spectrometer (Thermo Fisher Scientific, USA). Finally, an Xcalibur 2.3 software program (Thermo Fisher Scientific, USA) was used to assess QC. Chromatographic separation was done using a Waters ACQUITY UPLC BEH C18 Column at 45℃ (1.7 μm, 2.1 mm x 100 mm; Waters Corp., USA). The mobile phase was comprised of 0.1% formic acid (A) along with acetonitrile (B) in the positive mode, as well as 10 mM ammonium formate (A) along with acetonitrile (B) in the negative mode. The gradient conditions were as follows: 0–1 min, 2% B; 1–9 min, 2–98% B; 9–12 min, 98% B; 12–12.1 min, 98% B to 2% B; and 12.1–15 min, 2% B, adjusting the flow rate to 0.35 mL/min and using 5 µL injection volume.

The mass spectrometer was adjusted for positive/negative ionization modes as follows: 3.8/−3.2 kV spray voltage; 40 arbitrary units (arb) sheath gas flow rate; 10 arb aux gas flow rate; 350℃ aux gas heater temperature; and 320℃ capillary temperature. For MS acquisitions, the automatic gain control (AGC) target was 3e6 with a 100 ms maximum ion injection time, and the whole scan range was 70–1050 m/z at a 70,000 resolution. For subsequent MSMS fragmentation, the top three precursors were chosen with a 50 ms maximum ion injection time and 30,000 resolution, with 1e5 AGC. Collision energies were adjusted and stepped between 20, 40, and 60 Ev, with the interspersion of a QC sample every 10 samples.

### LC-MS/MS analysis

The method of LC-MS/MS analysis are described previously [15]. In brife, a Compound Discoverer 3.1 (Thermo Fisher Scientific, USA) was utilized to extract peaks, correct retention times, label background peaks, and identify metabolites from the raw LC-MS/MS data file. For each QC sample, the variation coefficient of the relative peak area was calculated, and the compounds with a variation coefficient above 30% were deleted. Metabolites were identified from various databases including the BGI Metabolome Database (BMDB), KEGG, and LIPID MAPS. The main metabolite identification parameters were as follows: Precursor Mass Tolerance < 5 ppm, Fragment Mass Tolerance < 10 ppm, and RT Tolerance < 0.2 min. Five levels of confidence were assigned to the metabolite identification levels, with an orderly reduction of credibility of levels 1 to 5.

### Plasma homocysteine (Hcy) analysis

Plasma samples (3 mL) were added into conical tubes and centrifuged at 1000 g at 4℃ for 15 min to remove cell fragments and lipids. The serum concentrations of Hcy were measured using a microparticle chemiluminescence immunoassay(MCIA) (ARCHITECT H0413R02A, USA) as previously described [16].

### Targeted GS-MS analysis

To validate the LC-MS/MS analysis, a subset of metabolites that were different between the groups in the study’s untargeted experiment was further assessed using the standard addition method. The UPLC-MS/MS system (ACQUITY UPLC-Xevo TQ-S) was utilized for targeted LC-MS analysis. Standards were acquired from Sigma-Aldrich (USA), Steraloids (USA), and TRC Chemicals (Canada). A total of 5.0 mg·mL − 1 individual stock solution was obtained by precisely weighing and preparing all the standards in an appropriate solution. Stock calibration solutions were created by mixing proper volumes of each stock solution. The preparation and measurement of the serum samples relied on earlier reported methods.

The mobile phases consisted of (A) 0.1% formic acid in water and (B) acetonitrile-methanol at a 70:30 ratio. Moreover, the ACQUITY BEH C18 Column (1.7 μm, 100 × 2.1 mm) (Waters) was injected with 5 µL of each sample at 40 °C; adjusting the flow rate at 0.40 mL·min − 1 with the mobile-phase gradient. The mass spectrometer was operated in the negative mode with a 2.0 kV capillary voltage and in the positive mode with a 1.5 kV capillary voltage. Temperatures were 150 °C at the source, and 550 °C at desolvation.

### Statistical analysis

- Values are reported as mean ± standard deviation (SD). The χ2 and Mann–Whitney test was used to compare clinical variables between groups. Welch’s t-test for unequal variances was utilized to test group differences for metabolites. Multivariate logistic regression analysis was conducted using the Guassian elimination method. Hcy metabolomic (HCY-Mtb) index was calculated as previously described [17]. Brifely, the selected metabolic variables(such as: Hcy, SAH and choline) were included as independent variables in the logistic regression analysis to generate regression coefficients(β) for each variable, and then the variables were multiplied by the regression coefficients to form a combined index regression equation.
- Area under receiver operating characteristic (ROC) curves were utilized to assess the indicators or risk model accuracy. The incremental predictive values were determined by performing the net reclassification improvement (NRI) and integrated differentiation improvement (IDI) risk models [18]. Results are represented as odds ratios (OR) with 95% confidence intervals (CI). A *P*-value < 0.05 or 0.01 (two-sided) indicated significant differences. Analyses were carried out using the SPSS package (IBM SPSS statistic 26, SPSS Inc) and R 4.2.1.

## Results

### Baseline characteristics of the participants in the CAD obese, Non-CAD obese, and Non-CAD lean groups

For the discovery cohorts, 62 participants were prospectively enrolled in the final analysis using a single standard protocol from January 2021 to May 2022. There were no significant differences in age, BMI (kg/m^2^), WC, hip circumference, hypertension, diabetes, uric acid, and serum lipids between the Non-CAD obese and the CAD obese groups, except for the history of smoking and male gender. Table 1 shows the general clinical characteristics of the three groups.

**Table 1:** Clinical characteristics of the participants in the CAD obese and Non-CAD obese groups in the validation cohorts

	CAD Obs (n = 23)	Non-CAD Obs (n = 22)	Lean control (n = 17)
Male, n(%)	20(86.90)*£	11(50.00)	5(29.40)
Age, years	54.10 ± 6.81	52.42 ± 7.40	55.46 ± 8.50
BMI(kg/m2)	29.20 ± 2.53£	30.40 ± 3.40	21.55 ± 1.50
Waist(cm)	101.50 ± 6.92£	103.30 ± 8.82	85.20 ± 6.52
Hip (cm)	102.90 ± 6.72£	104.20 ± 7.4	91.50 ± 7.02
Waist-to-hip ratio	0.98 ± 0.05	0.98 ± 0.06	0.94 ± 0.09
Hypertension, n (%)	13(56.50)	19(86.30)	7(41.10)
Diabetes, n (%)	10(43.40)£	5(22.70)	0(0.00)
Metabolic syndrome	12(52.17)£	14(60.86)	0(0.00)
Frequency of current smokers, n (%)	17(73.90)*£	6(27.20)	3(17.60)
Current alcohol, n(%)	3(13.00)	2(9.00)	0(0.00)
CAD family history, n(%)	4(17.30)	7(31.80)	1(5.80)
Hyperuricemia, n(%)	10(43.40)	16(72.70)	5(29.40)
Total cholesterol(TC), mg/dL	4.62 ± 1.24	4.59 ± 0.92	4.3 ± 1.00
HDL cholesterol,mg/dL	0.95 ± 0.16	1.02 ± 0.18	1.05 ± 0.26
LDL cholesterol, mg/dL	3.12 ± 1.10	2.83 ± 0.97	2.77 ± 0.87
Triglycerides(TG), mg/dL	1.93 ± 0.67	1.78 ± 1.27	1.49 ± 0.64

1. Data are shown as mean ± standard deviation unless noted otherwise

2. Differences in continuous variables among the groups were analyzed using one-way analysis of variance, and categorical data were analyzed using χ^2^ tests

3. *Significantly different from the Non-CAD obese group

### Metabolic pathways were the most differentially expressed pathways in the transcriptomics data analysis

**Fig. 1:**
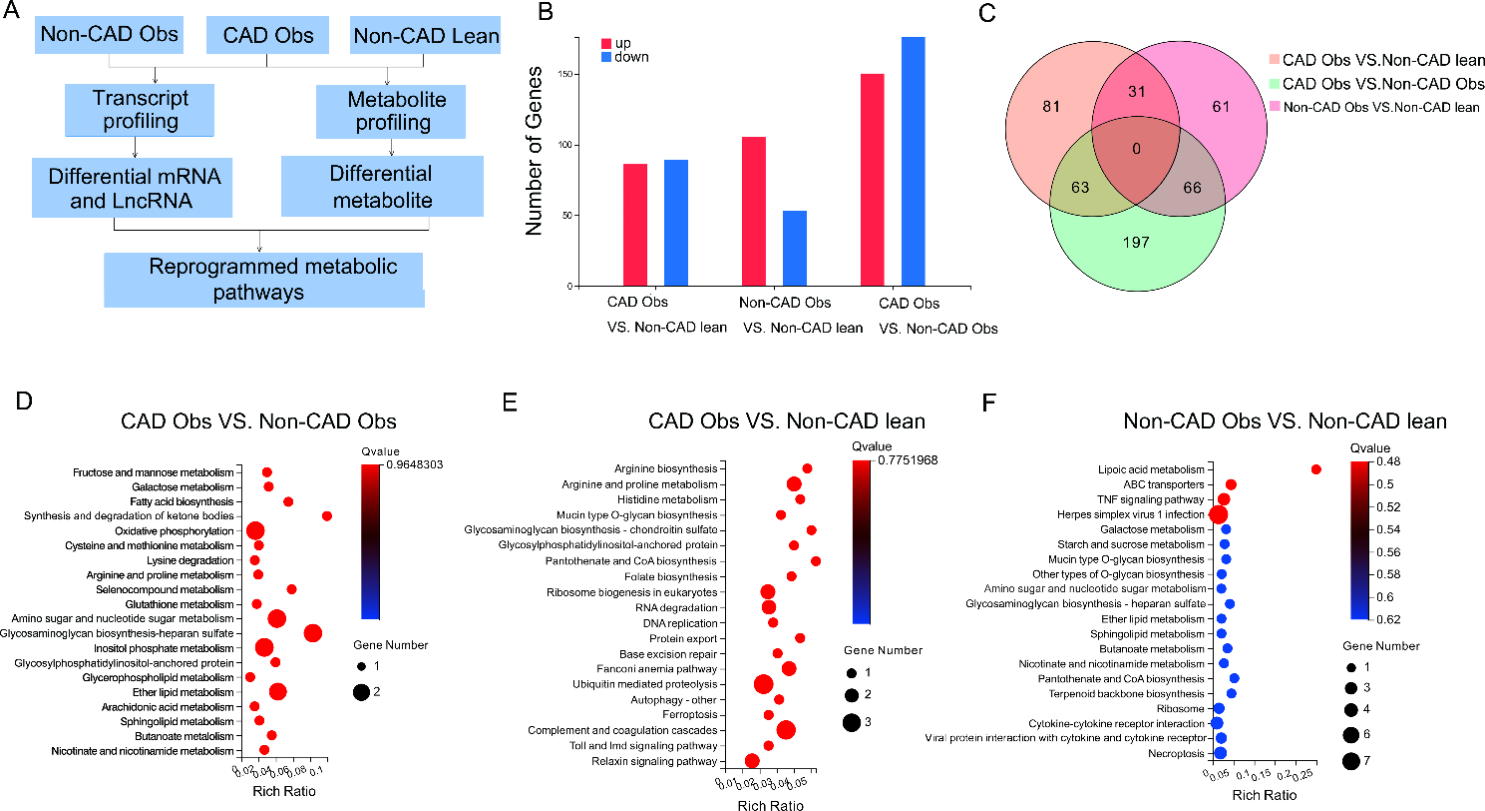
**Transcript profiling of the plasma deprived exosomal mRNA and LncRNA from the CAD obese, Non-CAD obese, and Non-CAD lean groups**. (**A**) Schematic diagram of the transcript and metabolomic study design; (**B**) The numbers of differentially expressed exosomal mRNAs and LncRNAs between the three groups; (**C**) Venn diagram of the three groups; (**D-F**) Top 20 altered canonical enriched KEGG pathways between the three groups. The size of the spot indicates the gene numbers in the enriched pathway, and the color indicates the significance level of the enriched pathway

The identification of isolated plasma-derived exosomes is shown in Figure S1. Differential expression analysis results showed that 272 mRNAs and 53 LncRNAs were identified as remarkably differentially expressed in the CAD obese group compared with the Non-CAD obese group. The numbers of differentially expressed genes between the three groups are shown in Fig. 1B, C.

As expected, pathway enrichment analysis of the Non-CAD obese and CAD obese groups revealed that the top 20 altered canonical KEGG pathways were mainly involved in fatty acid biosynthesis metabolism, cysteine and methionine metabolism, and arginine and proline metabolism (Fig. 1D-F). KEGG pathway analysis indicated that metabolic pathways were the most differentially expressed pathways between the groups. The next step will be to use metabolomics to identify differentially expressed metabolites.

### Untargeted metabolomic profiling of sera from CAD obese, Non-CAD obese, and Non-CAD lean groups

**Fig. 2:**
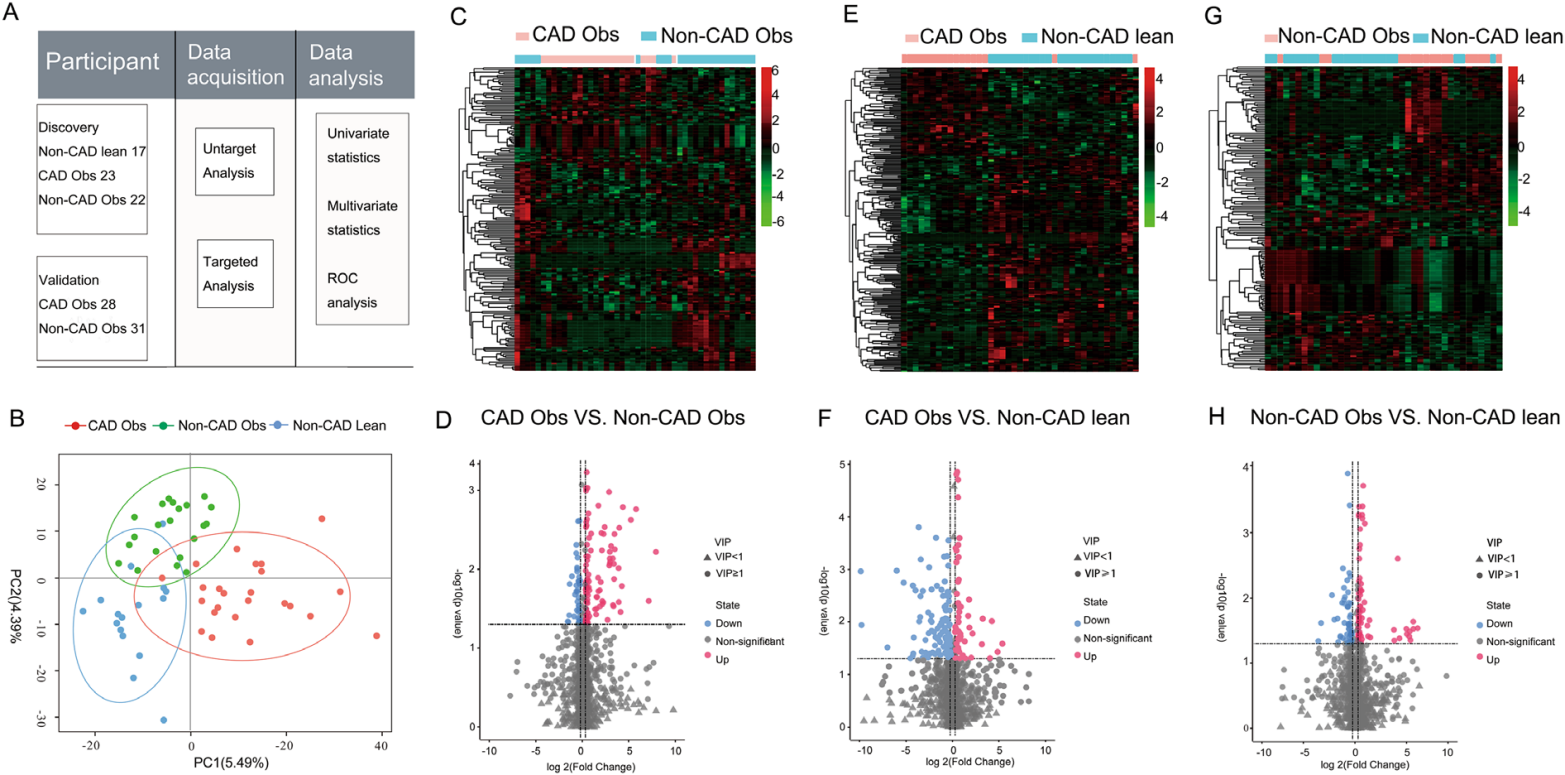
**Non-targeted metabolomics profiling of plasma from the CAD obese, Non-CAD obese, and Non-CAD lean groups in the discovery cohort**. (**A**) Schematic diagram of the study design; (**B**) PLS-DA of the untargeted metabolomics among the three groups; (**C**, **E** & **G**) Heatmap of the selected metabolites with a false discovery rate < 0.05 in the comparison of the CAD obese, Non-CAD obese, and Non-CAD lean groups, respectively; (**D**, **F** & **H**) Volcano plots highlighting the serum metabolites that were increased (red) or decreased (blue), with a false discovery rate < 0.05 and a log2 fold change > 0.25 or < − 0.25

Sixty-two serum samples were analyzed using LC-MS/MS in both the positive and negative ion modes. The significant aggregation of QC samples indicates good stability and repeatability based on principal component analysis (PCA) (Fig. S1). Figure 2 A shows a schematic diagram of the study design and Fig. 2B shows the results of the partial least squares discriminant analysis (PLS-DA) model. There was a clear distinction between the CAD obese, Non-CAD obese, and Non-CAD lean groups.

**Fig. 3:**
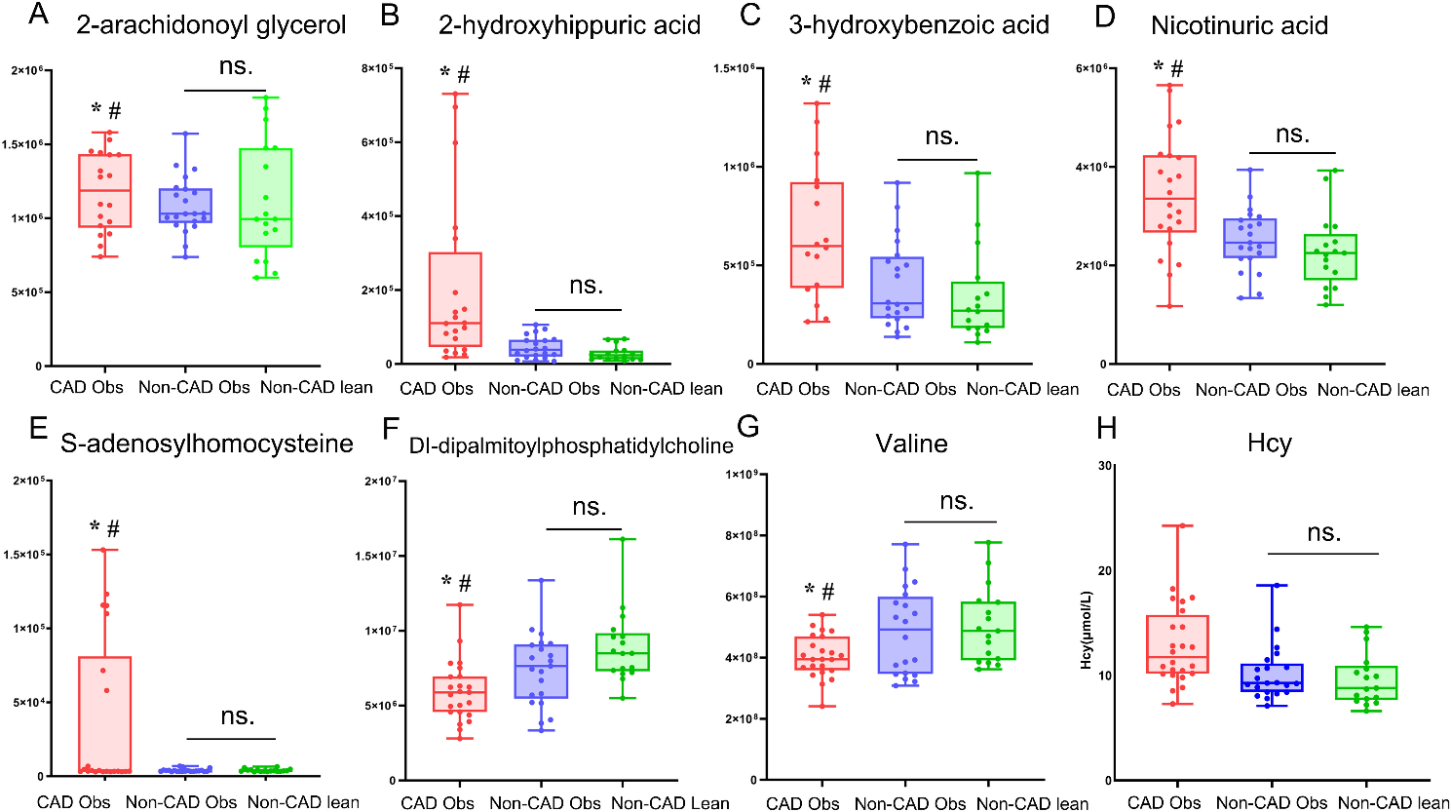
**Metabolites showing differences in the m/zRT intensities normalized to the internal standard in the CAD obese, Non-CAD obese, and Non-CAD lean control groups in the discovery cohort**. The box plot includes the median (horizontal line), and all points from the minimum and maximum data values, unless outliers are present; (**A**–**G**) Differences between the patients in the CAD obese, Non-CAD obese, and Non-CAD lean groups; * Significantly different from the Non-CAD obese group; # significantly different from the Non-CAD lean control group; ns: not significant. (**H**) Differences in the plasma Hcy levels tested using MCIA between patients in the CAD obese, Non-CAD obese, and Non-CAD lean groups

Metabolomics screening identified 4,139 peaks, of which 1,716 were named metabolites. Among them, 230 significantly differentially expressed metabolomics were detected in the plasma of the CAD obese and Non-CAD obese groups, with 154 upregulated and 76 downregulated metabolites (*P* < 0.05). A volcano map and heat map of the differentially expressed metabolites of the three groups are shown in Fig. 2C–H. Our results showed that SAH, 3-hydroxybenzoic acid, 2-hydroxyhippuric acid, nicotinuric acid, and 2-arachidonoyl glycerol were significantly elevated, and Dl-dipalmitoylphosphatidylcholine and valine were significantly reduced in the CAD obese group compared to the Non-CAD obese and Non-CAD lean groups, these above differentially expressed metabolites showed no significant difference between the Non-CAD obese and the Non-CAD lean groups shown in Fig. 3A–G. When adjusted for gender, smoking history, hypertension, and diabetes, multivariate logistic regression analysis showed 2-hydroxyhippuric acid, nicotinuric acid, and valine were significantly associated with CAD (*P* ≤ 0.05). The statistical analyses for the differentially expressed metabolites of the three groups are shown in Table S1.

### Plasma Hcy level significantly was increased in the CAD obese group

SAH and Hcy are important intermediate metabolites in the metabolic pathway of methionine. Because Hcy could not be detected in the untargeted metabonomics analyisis, serum concentrations of Hcy were measured using MCIA. The results showed that the level of Hcy in the CAD obese group was significantly more than Non-CAD obese and Non-CAD lean groups. However, no significant difference was detected between the other two groups (Fig. 3H).

### Cysteine and methionine metabolism were significantly altered at both the metabolomic and transcriptomics expression levels

**Fig. 4:**
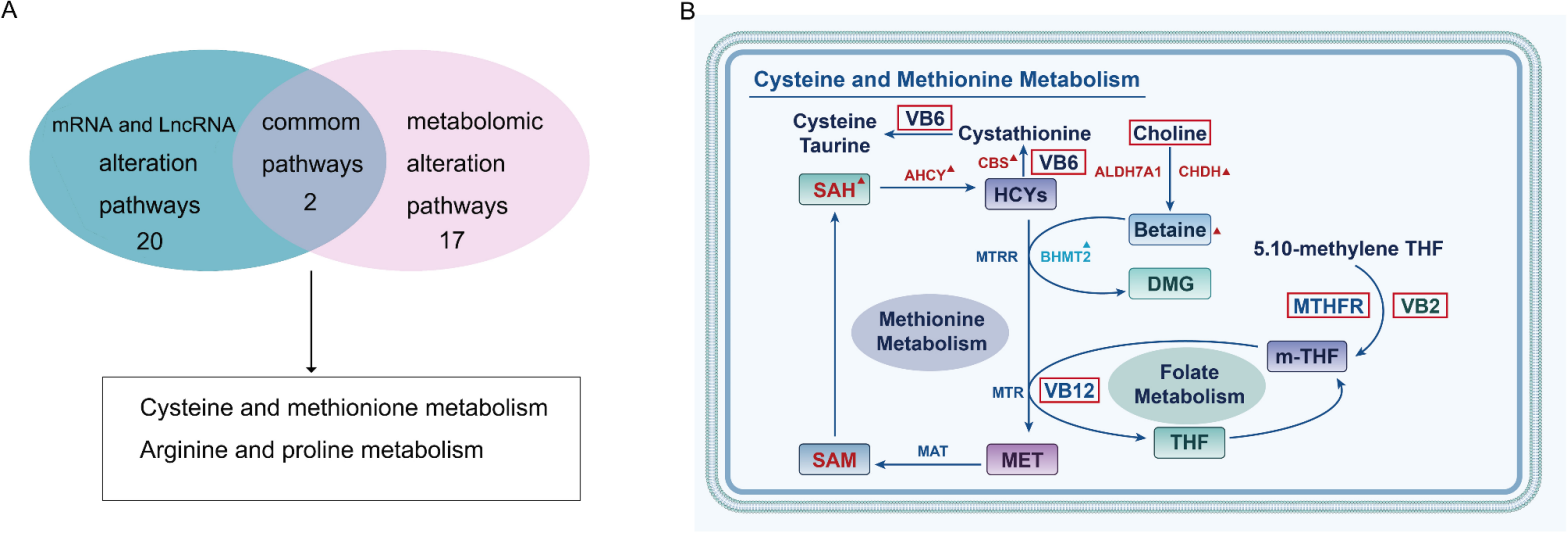
**Common alteration KEGG pathways at the metabolomic and transcriptomics expression**. (**A**) Cysteine and methionine metabolism in a common alteration pathway; (**B**) Schematic diagram of cysteine and methionine metabolism

When comparing the Non-CAD obese and CAD obese groups, KEGG pathway enrichment analysis revealed the enrichment of 17 canonical pathways that are involved in the biosynthesis of amino acids, cysteine and methionine metabolism, glycine, serine and threonine metabolism, cholesterol metabolism, arginine and proline metabolism, and bile secretion (Fig. S2). Two pathways were significantly altered at both the metabolomic and transcriptomics expression levels in the CAD obese and Non-CAD obese groups (Fig. 4A), including cysteine and methionine metabolism, and arginine and proline metabolism.

### Targeted metabolomic analysis revealed alterative metabolites in the CAD obese and Non-CAD obese groups

Hcy metabolic targeted metabonomics was next conducted to determine if cysteine and methionine metabolism pathway-related key molecules or nutrients were significantly different between the Non-CAD obese and CAD obese groups. A schematic diagram of cysteine and methionine metabolism is shown in Fig. 4B, and changes in choline, Hcy, SAH, 5-methylpropanedioic acid (MMA, related to Vitamin B12), betaine, S-adenosylmethionine (SAM), 4-Pyridoxic acid (4-PA, a product of vitamin B6), vitamin B2 (VB2), and 5-methyltetrahydrofolic acid (5-MTHF, active form of folic acid) were measured.

**Fig. 5:**
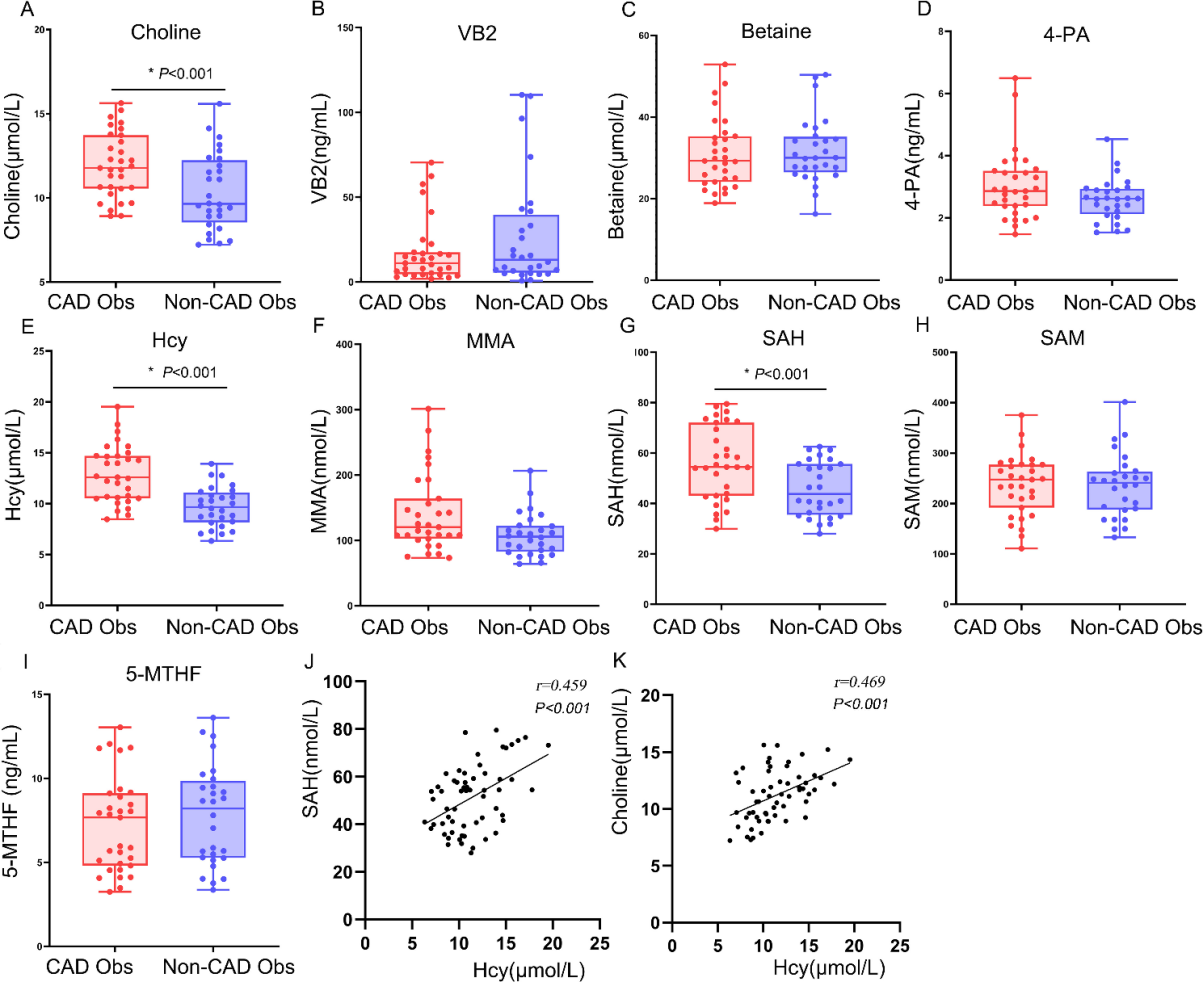
**Targeted metabolomics profiling of plasma from the CAD obese and the Non-CAD obese groups in the validation cohort**. The box plot includes the median (horizontal line), and all points from the minimum and maximum data values, unless outliers are present; (**A**–**I**) Differences between patients in the CAD obese and Non-CAD obese groups;. (**J**–**K**) *Person* correlation analysis was performed between plasma Hcy and SAH or choline

**Fig. 6:**
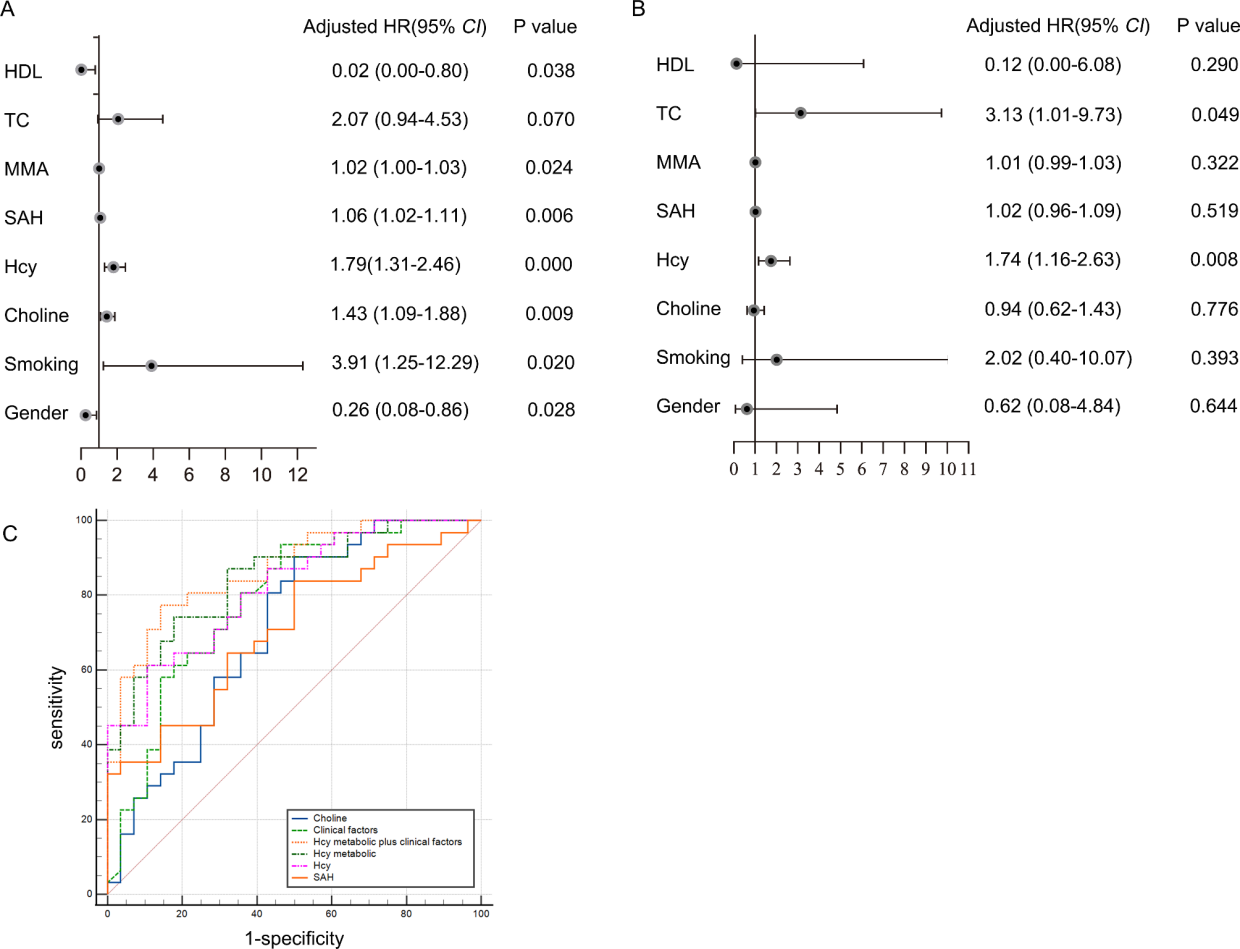
**Logistic regression and ROC analysis for targeted metabolomics profiling of plasma from the CAD obese and Non-CAD obese groups in the validation cohort**. (**A–B**) Univariate and multivariate logistic regression analysis showed that only Hcy was an independent predictor of obesity with CAD; (**C**) The AUC of models by Hcy, SAH, choline, or combinations with clinical characteristics were calculated separately

For the validation study, another 59 consecutive patients were prospectively enrolled independently from May 2022 to March 2023. Table 2 shows the basic clinical characteristics of the two groups. Only the basic characteristics of smoking history, creatinine, and HDL-C demonstrated significant differences between the two groups (*P* < 0.05). Targeted metabonomics showed that the levels of choline, Hcy, and SAH in the CAD obese group significantly increased, while MMA, betaine, SAM, 4-PA, VB2, 5-MTHF, and the SAM/SAH ratio were not significantly different between the two groups (Fig. 5A-I). In addition, *Person* correlation analysis showed that the levels of SAH and choline were positively correlated with Hcy in all obese participants (Fig. 5J–K). Univariable logistic regression analysis also demonstrated that smoking, male gender, HDL-C, choline, Hcy, SAH, and creatinine were statistically significant variables, and multivariate analysis showed that only Hcy was an independent predictor of obesity with CAD (OR:1.7; 95%CI:1.2–2.6; *P* = 0.008) after adjusting for smoking, gender, diabetes, and hypertension (Fig. 6A-B). CAD obese group had higher choline levels than the Non-CAD obese group, and that choline was positively associated with Hcy concentration. However, multivariate logistic regression analysis showed there was no significant association between choline and CAD(*P*>0.05).

**Table 2:** Clinical characteristics of participants in the CAD obese, Non-CAD obese, and Non-CAD lean groups in the discovery cohorts

	CAD Obs (n = 31)	Non-CAD Obs (n = 28)	P- value
Male, n(%)	24(77.40)	16(57.10)	0.96
Age, years	55.30 ± 6.25	53.50 ± 7.16	0.34
BMI(kg/m2)	29.50 ± 3.24	30.25 ± 2.70	0.79
Waist(cm)	101.50 ± 7.60	98.80 ± 7.60	0.29
Hip (cm)	103.30 ± 6.50	102.12 ± 8.00	0.64
Waist-to-hip ratio	0.98 ± 0.05	0.96 ± 0.04	0.32
Hypertension status, n (%)	22(70.90)	15(53.60)	0.17
Blood pressure, mmHg			
Systolic	135 ± 14	131 ± 9	0.56
Diastolic	85 ± 8	78.45 ± 8	0.45
Diabetes, n (%)	15(48.40)	8(28.50)	0.12
Frequency of current smokers, n (%)	16(51.60)*	6(21.40)	0.02
Current alcohol, n(%)	3(9.60)	3(10.70)	0.54
CAD family history, n(%)	7(22.60)	7(25.00)	0.83
Hyperuricemia, n(%)	14(45.10)	14(50.00)	0.71
Total cholesterol, mg/dL	4.50 ± 0.98	4.55 ± 0.96	0.83
HDL cholesterol,mg/dL	0.91 ± 0.16*	1.05 ± 0.31	0.01
LDL cholesterol, mg/dL	2.98 ± 0.84	2.93 ± 0.98	0.19
Triglycerides, mg/dL	1.84 ± 0.89	1.45 ± 0.62	0.06
Alanine aminotransferase (U/L)	28.23 ± 29.99	21.14 ± 11.35	0.24
HbAc1(%)	6.45 ± 1.51	6.28 ± 1.26	0.64
Creatinine(umol/L)	89.16 ± 19.01*	75.50 ± 16.14	0.00
Metabolic syndrome	21(67.74)	17(60.71)	0.68
Medical use			
Anti-platelet	12(38.70)	9(29.03)	0.78
Stains	10(32.25)	6(21.42)	0.39
β-blockers	18(58.06)	10(5.71)	0.12
AECI/ARB	19(61.29)	12(42.85)	0.19
Folic acid or VB12	0(0.00)	0(0.00)	-

1. Data are shown as mean ± standard deviation unless noted otherwise

2. Differences in the continuous variables among the groups were analyzed using one-way analysis of variance, and categorical data were analyzed using ×2 tests

3. *Significantly different from the Non-CAD obese group

Obesity is often accompanied by metabolic syndrome (MS), and MS significantly increases the risk of CAD [19]. To assess the effect of MS on the association between Hcy and CAD incidence, a separate model was fitted with MS×Hcy interaction terms. The diagnosis of MS was determined according to the ATP III classification [19]. The results showed there was no MS-Hcy interaction (*P* = 0.066).

### Combination of Hcy Metabolomic index and clinic variables improved the ability for discrimination and reclassification

The ROC curve showed that Hcy had an area under curve(AUC) of 0.819 (95%CI:0.715–0.923) for predicting CAD in obese patients. For a threshold of 10.67 µmoL/L, the sensitivity and specificity were 0.710 and 0.714, respectively, and the positive and negative predictive values were 0.830 and 0.600, respectively. To study the combined predictive value of the significant metabolites, the authors established a Hcy metabolomic index (HCY-Mtb.index) based on the linear combination of choline, Hcy, and SAH. The ROC AUC based on the HCY-Mtb.index alone was 0.840. Furthermore, the AUC improved from 0.791 for the most predictive clinical variables (smoking and male gender) to 0.877 for the clinical variables plus the Hcy-Mtb.index (Fig. 6C). And the inclusion of clinic risk factors in the Hcy metabolomic index model significantly improved the ability for discrimination and reclassification (category INR = 0.172, 95%*CI*: 0.028–0.315, *p* = 0.019; IDI = 0.062, 95%*CI*: 0.001–0.122, *p* = 0.046).

## Discussion

This is the first study, to our knowledge, that systematically provides a comprehensive view of metabolic characterization between Non-CAD obese and CAD Chinese obese individuals aged 40–65 years. Transcriptomics and metabolomics profiling indicated that the cysteine and methionine metabolism pathway is involved in the occurrence of CAD in obese individuals. The authors proposed clinical prediction models using the Hcy metabolic pathway and clinical data, providing a first-step approach to identify a subgroup of obese individuals at higher risk of developing CAD.

Our untargeted metabolomics analysis identified 230 differentially expressed metabolites between Non-CAD obese and CAD obese individuals compared to between Non-CAD obese and lean-healthy individuals, demonstrating a pronounced metabolic profiling shift when CAD occurs in obese patients. Among these differential metabolites, some were related to cardiovascular diseases, such as SAH, nicotinuric acid, and 2-AG. A previous study showed that higher SAH levels and a lower SAM:SAH ratio are better indicators of cardiovascular disease than Hcy [20, 21]. Our findings demonstrated a significant elevation in SAH, but no significant change in the SAM:SAH ratio in CAD obese patients. Several previous studies have shown that urine nicotinuric acid was higher in subjects with diabetes than in those without diabetes through a metabolomics-based approach. Thus, nicotinuric acid has been proposed to be a potential marker of metabolic syndrome [22, 23]. Another previous study reported that circulating 2-AG levels are positively associated with visceral fat and fasting glucose and increased in individuals with obesity and type 2 diabetes [24–26]. However, these above metabolites showed no significant difference between the Non-CAD obese and the Non-CAD lean control groups, which meant that these metabolites biomarkers might not be unique to obese population. It is necessary to introduce the forth group CAD lean to confirm these results, further research is needed to determine whether these metabolites causatively participate in the pathogenesis of CAD in obese patients.

Our pathway enrichment analysis of different exosome-derived small RNA and metabolites suggested a significant change in cysteine and methionine metabolism in the CAD obese group. Hcy, a intermediate product of cysteine and methionine metabolism, is an important indicator of cardiocerebrovascular diseases, and Hcy has been proposed to contribute to the formation of plaques by generating reactive oxygen species [27]. Several articles have reported the high Hcy levels increased the risk of CAD in obese individuals compared to non-obese individuals [28, 29], and higher Hcy levels were eleveated in obese subjects with hyperinsulinemic or metabolic syndrome compared to those without [30, 31]. But few studies have assessed the effects of changes in Hcy levels and the risk of developing CAD within different obese subgroups. This is the first study showing that serum Hcy levels are significantly elevated in CAD obese individuals compared with Non-CAD obese individuals, and that every 1 umol/L rise in Hcy is linked to a 74% increase in the risk of CAD in obese individuals, however the relationship between Hcy and CAD did not vary in the presence of metabolic syndrome. Similarly, a prior study showed that each additional 5 µmol/L increase in Hcy was associated with an approximate 20% increase in cardiovascular events [32]. In addition, there was no significant difference in Hcy and SAH levels between the Non-CAD obese and Non-CAD lean groups in this study, suggesting that not all of obese individuals exhibit an increase in serum Hcy levels. These findings are consistent with prior studies in which the plasma Hcy and SAH levels in obese subjects without atherosclerosis and impaired renal function did not differ from healthy subjects [33]. Thus, Hcy screening may be useful to exclude the possibility of CAD and avoid additional unnecessary tests for obese individuals with or without diabetes and hypertension.

The Hcy metabolic pathway depends on the proper functioning of methylene, tetrahydrofolate reductase enzyme, VB2, vitamin6 (VB6), vitamin12 (VB12), choline, betaine, and folic acid, which are all closely associated with circulating Hcy levels and cardiovascular and cerebrovascular events [34–37]. However a previous meta-analysis showed no evidence to support the use of Hcy-lowering interventions (including folate, betaine, and B-complex vitamins:VB2, VB6, and VB12) to prevent cardiovascular events [38]. Therefore, prescription of these interventions is not justified, and the relationship between these factors and hyperhomocysteinemia in CAD obese patients requires further research.

Our findings showed that plasma Hcy levels were significantly elevated, but there were no significant changes in 5-MTHF, betaine, VB2, and 4-PA in the CAD obese group compared with the Non-CAD obese group. In parallel, there was no linear correlation between these factors and Hcy levels in the obese participants in our study. It is interesting that the CAD obese group had higher choline levels than the Non-CAD obese group, and that choline was positively associated with Hcy concentration. However, multivariate logistic regression analysis showed that it was not significantly associated with CAD (*P*>0.05), further research is needed to increase the sample size to determine whether choline act as a risk factor. These results suggest that CAD obese patients with hyperhomocysteinemia may not necessarily present with folic acid, betaine, and complex vitamin B deficiency, and supplementation with these nutritions may have little to no benefit. Thus, other underlying mechanisms leading to hyperhomocysteinemia should be investigated. Although recent findings pointed towards new novel pharmacological targets to lower Hcy levels in vitro [39, 40], further investigation is warranted to confirm the beneficial effect on human subjects.

Only a few studies have assessed a risk model for predicting cardiovascular complications in different obese subgroups. Isabelle and colleagues reported that elevated TG levels increased the risk of CAD in obese individuals with a WC > 90 cm (with an OR of 2.4) [8]. However, the present study found that the TG level did not predict CAD in obese individuals, probably owing to the small sample size and different study populations. The study did reveal that increased Hcy levels predicted CAD in obese patients with high accuracy (0.82). Hyperhomocysteinemia is defined as > 15 µmol/L, but an increasing number of studies suggest that Hcy levels are still associated with adverse cardiovascular events even within the normal plasma range, and meta-analysis showed that serum Hcy levels > 8 umol/L led to the development of atheromatous plaque [41]. Our study demonstrated that the cutoff point for hyperhomocysteinemia to predict CAD was 10.67 µmol/L, which had an optimum sensitivity and specificity of 71.0% and 71.4%, respectively. The Hcy-Mtb.index (combined with choline, Hcy, and SAH) slightly improved the predictive power. When the Hcy-Mtb.index combined with smoking and male gender, the diagnostic accuracy significantly increased to 0.88, which could be used as an inexpensive screening model to identify obsess patients at high risk for developing CAD.

This prospective study has several limitations that should be acknowledged. First, transcriptomics analysis showed many of these KEGG pathways only involved a few genes and many of Q values were>0.05, this was possibly because the numbers of exosome differentially genes between groups were relatively small, we need to increase the sample size to confirm these results. However the transcriptomics results still revealed some useful clues that pathways related to metabolism and diseases were most frequently affected. Second, the study cohort was small and larger cohorts are warranted. Despite this, the high consistency of the results from the discovery and validation studies supports the findings. Third, the cohorts had some important characteristics that were not matched. For example, in the validation cohorts, there were significant differences between the groups regarding smoking history, and previous studies demonstrated smoking can elevate Hcy and reduce folate levels in humans [42]. This creates a potential bias that should be considered when interpreting results. Nevertheless, after adjusting for smoking history, Hcy was still an independent risk factor for CAD in obese individuals.

## Conclusion

Considerable heterogeneity exists among the different subtypes of obesity, which has a significant impact on prognosis. It is essential to understand the different metabolic phenotypes of obese patients. We discovered that cysteine and methionine metabolism might be involved in the pathogenesis of obesity complicated with CAD, and obesity with hyperhomocysteinemia may be a useful intermediate metabolism phenotype to screen obese patients who are at high risk for developing CAD. This study demonstrated that folic acid, betaine, VB2, and VB6 had no significant correlation with hyperhomocysteinemia in CAD patients with obesity. Thus, there is an urgent need to identify novel therapeutic targets to lower elevated plasma Hcy levels in obese patients.

### Electronic supplementary material

Below is the link to the electronic supplementary material.

Supplementary Material 1

Supplementary Material 2

Supplementary Material 3

Supplementary Material 4

Supplementary Material 5

Supplementary Material 6

Supplementary Material 7

## Data Availability

The datasets used and analyzed during the present study are available from the corresponding author upon reasonable request.

## List of Abbreviations

2-AG: 2-arachidonoyl glycerol
4-PA: 4-Pyridoxic acid
5-MTHF: 5-Methyltetrahydrofolic acid
AUC: Area under curve
BMI: Body mass index
CAD: Coronary artery disease
Hcy: Homocysteine
HCY-Mtb.index: Hcy metabolomic index
HDL-C: High-density lipoprotein cholesterol
IDI: Integrated differentiation improvement
LC: Liquid chromatography
MMA: Methylpropanedioic acid
MS: Tandem mass spectrometry
NGS: Next-generation DNA sequencing
Non-CAD obese: Non-CAD patients with obesity
NRI: Net reclassification improvement
OR: Odds ratio
PCA: Principal component analysis
RNA-seq: RNA sequencing
ROC: Receiver operating characteristic
SAH: Adenosylhomocysteine
SAM: S-adenosylmethionine
SD: Standard deviation
TG: Fasting triglyceride
WC: Waist circumference

## References

[1] Powell-Wiley TM, Poirier P, Burke LE, Després JP, Gordon-Larsen P, Lavie CJ (2021). Obesity and cardiovascular disease: a scientific statement from the american heart association. Circulation.

[2] Manoharan MP, Raja R, Jamil A, Csendes D, Gutlapalli SD, Prakash K (2022). Obesity and coronary artery disease: an updated systematic review 2022. Cureus.

[3] Iacobini C, Pugliese G, Blasetti Fantauzzi C, Federici M, Menini S (2019). Metabolically healthy versus metabolically unhealthy obesity. Metabolism.

[4] Mayoral LP, Andrade GM, Mayoral EP, Huerta TH, Canseco SP, Rodal Canales FJ (2020). Obesity subtypes, related biomarkers & heterogeneity. Indian J Med Res.

[5] Smith GI, Mittendorfer B, Klein S (2019). Metabolically healthy obesity: facts and fantasies. J Clin Investig.

[6] Hamer M, Stamatakis E (2012). Metabolically healthy obesity and risk of all-cause and cardiovascular disease mortality. J Clin Endocrinol Metab.

[7] Hwang YC, Hayashi T, Fujimoto WY, Kahn SE, Leonetti DL, McNeely MJ (2015). Visceral abdominal fat accumulation predicts the conversion of metabolically healthy obese subjects to an unhealthy phenotype. Int J Obes (Lond).

[8] Lemieux I, Pascot A, Couillard C, Lamarche B, Tchernof A, Alméras N (2000). Hypertriglyceridemic waist: a marker of the atherogenic metabolic triad (hyperinsulinemia; hyperapolipoprotein B; small, dense LDL) in men?. Circulation.

[9] Ozsolak F, Milos PM (2011). RNA sequencing: advances, challenges and opportunities. Nat Rev Genet.

[10] Caroline H, Johnson J, Ivanisevic, Gary Siuzdak (2016). Metabolomics: beyond biomarkers and towards mechanisms. Nat Rev Mol Cell Biol.

[11] McGarrah RW, Crown SB, Zhang GF, Shah SH, Newgard CB (2018). Cardiovascular metabolomics. Circ Res.

[12] Zeng Q, He Y, Dong S, Zhao X, Chen Z, Song Z (2014). Optimal cut-off values of BMI, waist circumference and waist:height ratio for defining obesity in chinese adults. Br J Nutr.

[13] Mantella LE, Colledanchise KN, Hétu MF, Feinstein SB, Abunassar J, Johri AM (2019). Carotid intraplaque neovascularization predicts coronary artery disease and cardiovascular events. Eur Heart J Cardiovasc Imaging.

[14] Kim D, Langmead B, Salzberg SL. HISAT: a fast spliced aligner with low memory requirements. Nat Methods.2015;12, 357 – 60. 10.1038/nmeth.3317 PMC4655817 25751142

[15] Jia H, Liu C, Li D, Huang Q, Liu D, Zhang Y (2022). Metabolomic analyses reveal new stage-specific features of COVID-19. Eur Respir J.

[16] Kunanusont N, Punyadarsaniya D, Ruenphet S (2021). Accuracy and precision guidelines for optimal breeding time in bitches using in-house progesterone measurement compared with chemiluminescent microparticle immunoassay. Vet World.

[17] Haukka JK, Sandholm N, Forsblom C, Cobb JE, Groop PH, Ferrannini E (2018). Metabolomic Profile Predicts Development of Microalbuminuria in individuals with type 1 diabetes. Sci Rep.

[18] Lin XL, Li QY, Zhao DH, Liu JH, Fan Q (2022). Serum glycated albumin is associated with in-stent restenosis in patients with acute coronary syndrome after percutaneous coronary intervention with drug-eluting stents: an observational study. Front Cardiovasc Med.

[19] Engin A (2017). The definition and prevalence of obesity and metabolic syndrome. Adv Exp Med Biol.

[20] Wagner C, Koury MJ (2007). S-Adenosylhomocysteine: a better indicator of vascular disease than homocysteine?. Am J Clin Nutr.

[21] Xiao J, You Y, Chen X, Tang Y, Chen Y, Liu Q (2022). Higher S-adenosylhomocysteine and lower ratio of S-adenosylmethionine to S-adenosylhomocysteine were more closely associated with increased risk of subclinical atherosclerosis than homocysteine. Front Nutr.

[22] Pfuhl P, Kärcher U, Häring N, Baumeister A, Tawab MA, Schubert-Zsilavecz M (2005). Simultaneous determination of niacin, niacinamide and nicotinuric acid in human plasma. J Pharm Biomed Anal.

[23] Huang CF, Cheng ML, Fan CM, Hong CY, Shiao MS (2013). Nicotinuric acid: a potential marker of metabolic syndrome through a metabolomics-based approach. Diabetes Care.

[24] Blüher M, Engeli S, Klöting N, Berndt J, Fasshauer M, Bátkai S (2006). Dysregulation of the peripheral and adipose tissue endocannabinoid system in human abdominal obesity. Diabetes.

[25] Engeli S, Böhnke J, Feldpausch M, Gorzelniak K, Janke J, Bátkai S (2005). Activation of the peripheral endocannabinoid system in human obesity. Diabetes.

[26] Matias I, Gonthier MP, Orlando P, Martiadis V, De Petrocellis L, Cervino C (2006). Regulation, function, and dysregulation of endocannabinoids in models of adipose and beta-pancreatic cells and in obesity and hyperglycemia. J Clin Endocrinol Metab.

[27] Cheng CK, Luo JY, Lau CW, Cho WC, Ng CF, Ma RCW (2021). A GLP-1 analog lowers ER stress and enhances protein folding to ameliorate homocysteine-induced endothelial dysfunction. Acta Pharmacol Sin.

[28] Yologlu S, Sezgin AT, Sezgin N, Ozdemir R, Yesilada E, Topal E (2005). Determination of risk factors in obese and non-obese patients with coronary artery disease. Acta Cardiol.

[29] Kouzehgaran S, Vakili R, Nematy M, Safarian M, Ghayour-Mobarhan M, Khajedaluee M (2015). Comparison of novel coronary artery disease risk factors between obese and normal adolescent. Iran J Med Sci.

[30] Sreckovic B, Sreckovic VD, Soldatovic I, Colak E, Sumarac-Dumanovic M, Janeski H (2017). Homocysteine is a marker for metabolic syndrome and atherosclerosis. Diabetes Metab Syndr.

[31] Sanchez-Margalet V, Valle M, Ruz FJ, Gascon F, Mateo J, Goberna R (2002). Elevated plasma total homocysteine levels in hyperinsulinemic obese subjects. J Nutr Biochem.

[32] Humphrey LL, Fu R, Rogers K, Freeman M, Helfand M (2008). Homocysteine level and coronary heart disease incidence: a systematic review and meta-analysis. Mayo Clin Proc.

[33] Uysal O, Arikan E, Cakir B (2005). Plasma total homocysteine level and its association with carotid intima-media thickness in obesity. J Endocrinol Invest.

[34] Chrysant SG, Chrysant GS (2018). The current status of homocysteine as a risk factor for cardiovascular disease: a mini review. Expert Rev Cardiovasc Ther.

[35] Van Parys A, Brække MS, Karlsson T, Vinknes KJ, Tell GS, Haugsgjerd TR (2022). Assessment of dietary choline intake, contributing food items, and associations with one-carbon and lipid metabolites in middle-aged and elderly adults: the hordaland health study. J Nutr.

[36] Ashtary-Larky D, Bagheri R, Ghanavati M, Asbaghi O, Tinsley GM, Mombaini D (2022). Effects of betaine supplementation on cardiovascular markers: a systematic review and Meta-analysis. Crit Rev Food Sci Nutr.

[37] Angelini A, Cappuccilli ML, Magnoni G, Croci Chiocchini AL, Aiello V, Napoletano A et al. The link between homocysteine, folic acid and vitamin B12 in chronic kidney disease. G Ital Nefrol. 2021;38. 34469084

[38] Martí-Carvajal AJ, Solà I, Lathyris D (2015). Homocysteine-lowering interventions for preventing cardiovascular events. Cochrane Database Syst Rev.

[39] Li J, Luo M, Xie N, Wang J, Chen L (2016). Curcumin protects endothelial cells against homocysteine induced injury through inhibiting inflammation. Am J Transl Res.

[40] Chen J, Huang Y, Hu X, Bian X, Nian S (2021). Gastrodin prevents homocysteine-induced human umbilical vein endothelial cells injury via PI3K/Akt/eNOS and Nrf2/ARE pathway. J Cell Mol Med.

[41] Clarke R, Bennett DA, Parish S, Verhoef P, Dötsch-Klerk M, Lathrop M (2012). Homocysteine and coronary heart disease: meta-analysis of MTHFR case-control studies, avoiding publication bias. PLoS Med.

[42] Haj Mouhamed D, Ezzaher A, Neffati F, Douki W, Najjar MF (2011). Effect of cigarette smoking on plasma homocysteine concentrations. Clin Chem Lab Med.

