# Supplementary figures and images for: Comprehensive transcriptomics and metabolomics analyses reveal that hyperhomocysteinemia is a high risk factor for coronary artery disease in a chinese obese population aged 40–65: a prospective cross-sectional study
Source: Cardiovasc Diabetol. 2023 Aug 24;22:219. doi: 10.1186/s12933-023-01942-0 (PMC10463368; doi:10.1186/s12933-023-01942-0)

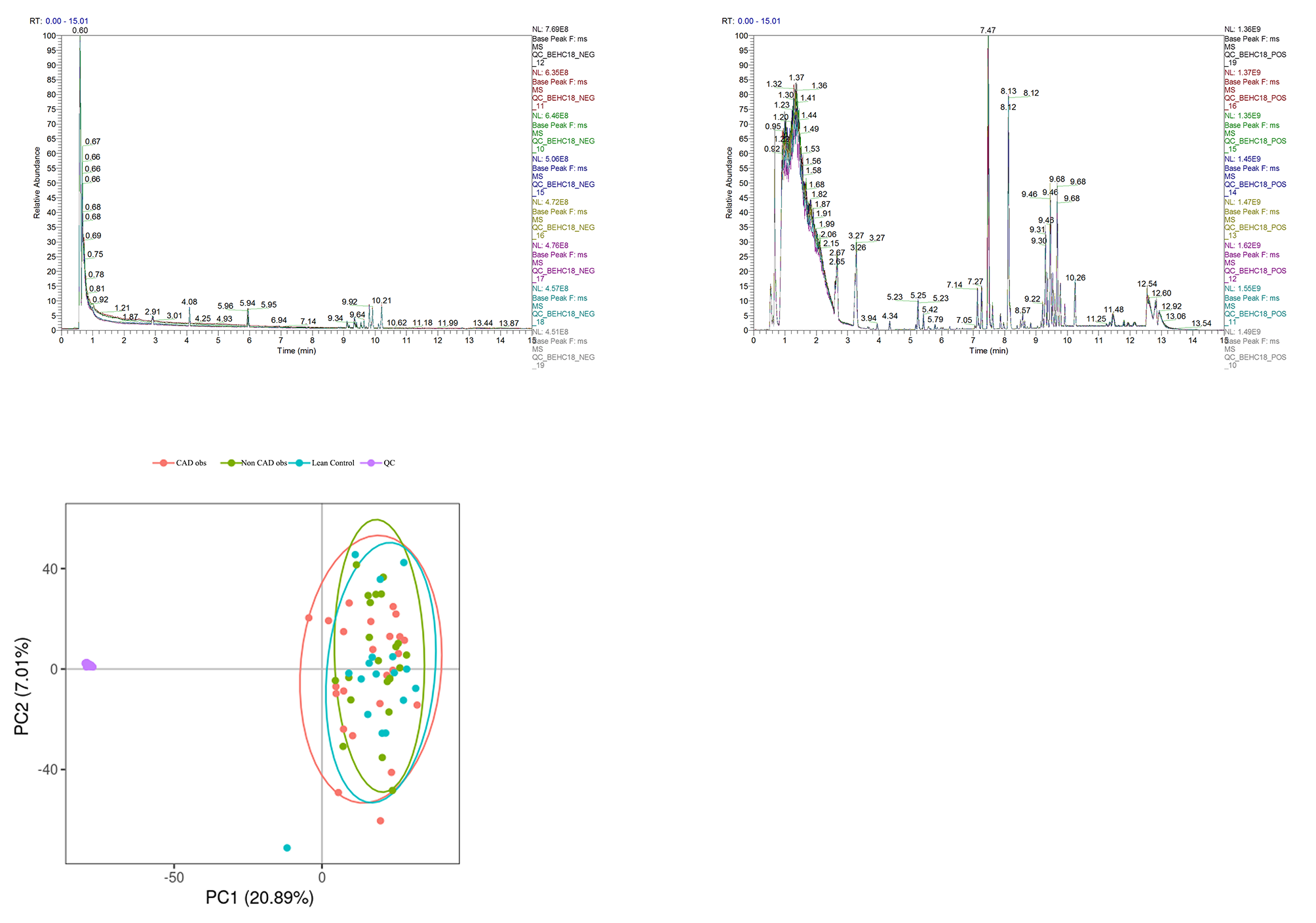

Supplement: Supplementary file 1 — Supplementary Material 1 [file 12933_2023_1942_MOESM1_ESM.png]

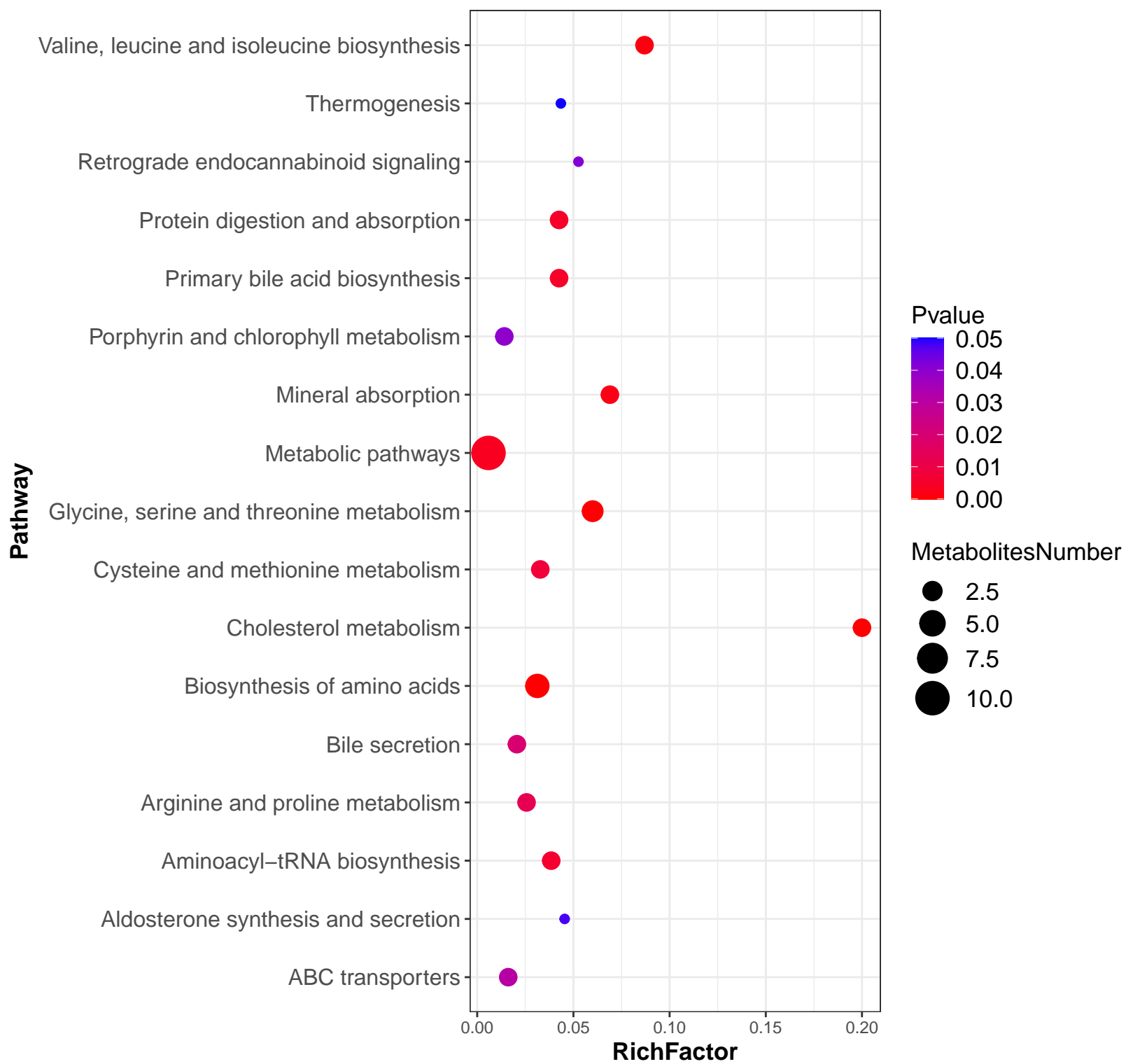

Supplement: Supplementary file 2 — Supplementary Material 2 [file 12933_2023_1942_MOESM2_ESM.pdf]
