# Supplementary material for: Comprehensive transcriptomics and metabolomics analyses reveal that hyperhomocysteinemia is a high risk factor for coronary artery disease in a chinese obese population aged 40–65: a prospective cross-sectional study
Source: Cardiovasc Diabetol. 2023 Aug 24;22:219. doi: 10.1186/s12933-023-01942-0 (PMC10463368; doi:10.1186/s12933-023-01942-0)
Supplement: Supplementary file 3 — Supplementary Material 3 [file 12933_2023_1942_MOESM3_ESM.docx]

| Mode | Group | Total number of  differential metabolites | up | down |
| --- | --- | --- | --- | --- |
| pos | Lean control VS CAD obese | 261 | 76 | 185 |
| pos | Lean control VS Non-CAD obese | 179 | 85 | 94 |
| pos | CAD obese VS Non-CAD obese | 192 | 128 | 64 |
| neg | Lean control VS CAD obese | 48 | 19 | 29 |
| neg | Lean control VS Non-CAD obese | 33 | 22 | 11 |
| neg | CAD obese VS Non-CAD obese | 38 | 26 | 12 |

Supplementary table 1 Statistics of differential metabolites for different group
