# Supplementary material for: Comprehensive transcriptomics and metabolomics analyses reveal that hyperhomocysteinemia is a high risk factor for coronary artery disease in a chinese obese population aged 40–65: a prospective cross-sectional study
Source: Cardiovasc Diabetol. 2023 Aug 24;22:219. doi: 10.1186/s12933-023-01942-0 (PMC10463368; doi:10.1186/s12933-023-01942-0)
Supplement: Supplementary file 4 — Supplementary Material 4 [file 12933_2023_1942_MOESM4_ESM.docx]

| Name | type | Average TPM | | | Log2 fold change | | | *P* value | | |
| --- | --- | --- | --- | --- | --- | --- | --- | --- | --- | --- |
|  |  | Non-CAD Obs | CAD  Obs | Non-CAD lean | Non-CAD lean  VS  CAD obs | Non-CAD lean  VS  Non-CAD obs | CAD obs  VS  Non-CAD Obs | Non-CAD lean  VS  CAD obs | Non-CAD lean  VS  Non-CAD obs | CAD obs  VS  Non-CAD Obs |
| TMOD2 | mRNA | 0 | 80.56 | 2.13 | -7.65 | 1.72 | 9.38 | 0.0066 | 0.37 | 0.000758 |
| GSTK1 | mRNA | 0 | 77.85 | 1.21 | -4.24 | 4.42 | 22.02 | 0.24 | 0.12 | 2.7E-10 |
| HSPB11 | mRNA | 0 | 131.66 | 0.53 | -3.53 | 4.89 | 21.79 | 0.37 | 0.1 | 3.2E-10 |
| STX8 | mRNA | 0 | 214.14 | 11.58 | -5.5 | 3.88 | 9.38 | 0.1 | 0.09 | 0.000859 |
| EIF1AX | mRNA | 0.07 | 32.16 | 0.89 | -5.28 | 3.36 | 8.65 | 0.05 | 0.08 | 0.00109 |
| USP47 | mRNA | 7.56 | 0.08 | 1.04 | 1.18 | -6.09 | -7.25 | 0.54 | 0.00334 | 0.00104 |
| IRF2BPL | mRNA | 15.59 | 0 | 0 | 8.72 | NA | -9.7 | 0.00233 | NA | 0.000994 |
| WASF3 | mRNA | 19.14 | 0 | 0.2 | 4.25 | -6 | -10.24 | 0.08 | 0.06 | 0.000582 |
| RIPOR1 | mRNA | 24.93 | 0 | 0.13 | 5.31 | -4.59 | -9.9 | 0.02 | 0.21 | 0.000485 |
| DDX10 | mRNA | 29.28 | 0 | 0.03 | 6.9 | -3.12 | -10.07 | 0.00525 | 0.44 | 0.000301 |
| LANCL1 | mRNA | 32.79 | 0.13 | 0.02 | 8.11 | -0.12 | -8.25 | 0.00138 | 0.98 | 0.00135 |
| PLRG1 | mRNA | 78.53 | 0 | 0.58 | 4.04 | -4.97 | -9.03 | 0.11 | 0.2 | 1.49E-03 |
| GFM2 | mRNA | 106.46 | 0.13 | 0.3 | 8.01 | -1.68 | -9.67 | 3.42E-03 | 0.67 | 6.34E-04 |
| NDUFS4 | mRNA | 517.63 | 0 | 17.47 | 3.41 | -7.93 | -11.35 | 0.1 | 3.04E-03 | 7.02E-05 |
| FBXO30-DT | lncRNA | 0 | 11.72 | 0 | NA | 20.93 | 21.12 | NA | 2.00E-09 | 2.00E-08 |
| BGIG9606_92140 | lncRNA | 0 | 13.33 | 0 | NA | 20.7 | 20.89 | NA | 3.00E-09 | 2.00E-08 |
| SMURF2P1-LRRC37BP1 | lncRNA | 0 | 7.31 | 0 | NA | 20.47 | 20.85 | NA | 4.00E-09 | 2.00E-08 |
| LOC107985423 | lncRNA | 0 | 8.56 | 0 | NA | 20.66 | 20.86 | NA | 3.00E-09 | 2.00E-08 |
| BGIG9606_76506 | lncRNA | 0 | 8.15 | 0 | NA | 20.45 | 20.71 | NA | 4.00E-09 | 3.00E-08 |
| LOC107984703 | lncRNA | 0 | 21.94 | 0 | NA | 20.99 | 20.88 | NA | 2.00E-09 | 3.00E-08 |

Supplementary table 2 Representative differentially expressed mRNA and LncRNA for different group comparision.
