# Supplementary material for: Comprehensive transcriptomics and metabolomics analyses reveal that hyperhomocysteinemia is a high risk factor for coronary artery disease in a chinese obese population aged 40–65: a prospective cross-sectional study
Source: Cardiovasc Diabetol. 2023 Aug 24;22:219. doi: 10.1186/s12933-023-01942-0 (PMC10463368; doi:10.1186/s12933-023-01942-0)
Supplement: Supplementary file 5 — Supplementary Material 5 [file 12933_2023_1942_MOESM5_ESM.docx]

| Name | Group comparision | ratio | t.test_p.value | LC platform | level | VIP | Actual.RT |
| --- | --- | --- | --- | --- | --- | --- | --- |
| 3-hydroxybenzoic acid | CAD obs VS Non-CAD Obs | 2.7819 | 0.0298 | C18:neg | level2 | 1.9272 | 2.932 |
| 3-hydroxybenzoic acid | Non-CAD lean VS CAD obs | 0.4661 | 0.0111 | C18:neg | level2 | 2.1198 | 2.932 |
| 3-hydroxybenzoic acid | Non-CAD lean VS Non-CAD obs | 1.2965 | 0.5166 | C18:neg | level2 | 0.6021 | 2.932 |
|  |  |  |  |  |  |  |  |
| 2-hydroxyhippuric acid | CAD obs VS Non-CAD Obs | 3.7131 | 0.0137 | C18:neg | level2 | 2.2545 | 3.376 |
| 2-hydroxyhippuric acid | Non-CAD lean VS CAD obs | 0.1206 | 0.0021 | C18:neg | level2 | 2.6716 | 3.376 |
| 2-hydroxyhippuric acid | Non-CAD lean VS Non-CAD obs | 1.4503 | 0.3343 | C18:neg | level2 | 0.9537 | 3.376 |
|  |  |  |  |  |  |  |  |
| 1-oleoyl-rac-glycerol | CAD obs VS Non-CAD Obs | 12.7168 | 0.0044 | C18:pos | level2 | 2.8995 | 9.76 |
| 1-oleoyl-rac-glycerol | Non-CAD lean VS CAD obs | 0.071 | 0.0025 | C18:pos | level2 | 2.5773 | 9.76 |
| 1-oleoyl-rac-glycerol | Non-CAD lean VS Non-CAD obs | 0.9023 | 0.5577 | C18:pos | level2 | 0.4424 | 9.76 |
|  |  |  |  |  |  |  |  |
| 2-arachidonoyl glycerol | CAD obs VS Non-CAD Obs | 1.305 | 0.0452 | C18:pos | level2 | 1.5147 | 9.521 |
| 2-arachidonoyl glycerol | Non-CAD lean VS CAD obs | 0.783 | 0.057 | C18:pos | level2 | 1.2976 | 9.521 |
| 2-arachidonoyl glycerol | Non-CAD lean VS Non-CAD obs | 1.0308 | 0.8826 | C18:pos | level2 | 0.1742 | 9.521 |
|  |  |  |  |  |  |  |  |
| Lauroylcarnitine | CAD obs VS Non-CAD Obs | 1.2792 | 0.0645 | C18:pos | level2 | 1.063 | 7.771 |
| Lauroylcarnitine | Non-CAD lean VS CAD obs | 0.7095 | 0.0164 | C18:pos | level2 | 1.6181 | 7.771 |
| Lauroylcarnitine | Non-CAD lean VS Non-CAD obs | 0.9076 | 0.232 | C18:pos | level2 | 0.9959 | 7.771 |
|  |  |  |  |  |  |  |  |
| Nicotinuric acid | CAD obs VS Non-CAD Obs | 1.4112 | 0.017 | C18:pos | level2 | 1.3456 | 3.449 |
| Nicotinuric acid | Non-CAD lean VS CAD obs | 0.6333 | 0.0021 | C18:pos | level2 | 1.5847 | 3.449 |
| Nicotinuric acid | Non-CAD lean VS Non-CAD obs | 0.8938 | 0.1942 | C18:pos | level2 | 0.6578 | 3.449 |
|  |  |  |  |  |  |  |  |
| Dl-dipalmitoylphosphatidylcholine | CAD obs VS Non-CAD Obs | 0.7754 | 0.0285 | C18:pos | level2 | 1.6286 | 11.841 |
| Dl-dipalmitoylphosphatidylcholine | Non-CAD lean VS CAD obs | 1.4789 | 0 | C18:pos | level2 | 1.9329 | 11.841 |
| Dl-dipalmitoylphosphatidylcholine | Non-CAD lean VS Non-CAD obs | 1.1468 | 0.0941 | C18:pos | level2 | 0.9038 | 11.841 |
|  |  |  |  |  |  |  |  |
| S-adenosylhomocysteine | CAD obs VS Non-CAD Obs | 16.6823 | 0.0027 | C18:pos | level3 | 3.5143 | 5.605 |
| S-adenosylhomocysteine | Non-CAD lean VS CAD obs | 0.0594 | 0.0026 | C18:pos | level3 | 3.1497 | 5.605 |
| S-adenosylhomocysteine | Non-CAD lean VS Non-CAD obs | 0.9912 | 0.9279 | C18:pos | level3 | 0.2092 | 5.605 |
|  |  |  |  |  |  |  |  |
| Valine | CAD obs VS Non-CAD Obs | 0.777 | 0.008 | C18:pos | level2 | 1.2944 | 0.729 |
| Valine | Non-CAD lean VS CAD obs | 1.2746 | 0.0021 | C18:pos | lveve2 | 1.1228 | 0.729 |
| Valine | Non-CAD lean VS Non-CAD obs | 0.9904 | 0.8908 | C18:pos | level2 | 0.1687 | 0.729 |
|  |  |  |  |  |  |  |  |
| L-threonine | CAD obs VS Non-CAD Obs | 1.3627 | 0.0012 | C18:neg | level1 | 1.3902 | 0.668 |
| L-threonine | Non-CAD lean VS CAD obs | 0.9951 | 0.8594 | C18:neg | level1 | 0.0795 | 0.668 |
| L-threonine | Non-CAD lean VS Non-CAD obs | 1.3561 | 0.009 | C18:neg | level1 | 1.3488 | 0.668 |

Supplementary table 3 Representative differentially expressed metabolites for different group comparision.
